# 

**Published:** 1883-12-20

**Authors:** 


### TABLE OF CONTENTS.

ORIGINAL AND SELECTED ARTICLES.

Prof. T S. Powell's Lecture to the Medical Class at the Southern Medical College, Atlanta, Ga., Oct. 1883, 441. A Remarkable Case of Fibro-Cellular and Cartilaginous Tumor, by Jno Thad Johnson, M. D., Prof. of Principles and Practice of Surgery in the Southern Medical College, Atlanta, Ga., 448. Some Thoughts Concerning Old Remedies, etc., by Harvey L. Byrd, M.D., Baltimore, 450. On the So-called Equine Scarlatina, by Frank S. Billings, M.D., 453. On the Local Treatment of Erysipelas, by John Kent Spender, M.D., London, 454. Use of Apomorphia in Cases of Poisoning, by Amand Routh, M.D., London, 455. Bi-Chloride of Mercury in Diphtheria, by Madison Reece, M.D., Abingdon, Ill., 457. On the Removal of the Placenta, 458.

ABSTRACTS AND GLEANINGS.

Tartar-Emetic in Skin Affections, 459. New Operation for Prolapsus Recti, 460. The Physician, 461. Specific Treatment of Typhoid Fever; Glycerine, 462. The Antagonism between Paraldehyde and Strychnine; Croup and Diphtheria, 463. Treatment of Typhoid Fever, 464. Benzoate of Iodine; Auto-Transfusion in Hemorrhage, 465. Syphilitic Inoculation of Monkeys; Cascara Amarga, 466. Petroleum in Phthisis—Consumption, 467. Treatment of Puerperal Convulsions, 468. Hot Milk as a Restorative; New Code, 469. Hæmoptysis; To Remove Fish Bones from the Throat; Small-pox—Petroleum as an Ectrotic, 470.

SCIENTIFIC ITEMS.

Gas Poisoning; Metallized Wood, 471. Climate and Character; Protective Coating for Glass, 472.

PRACTICAL NOTES AND FORMULÆ.

Harness Polish; Creasote Wine; Iodoform in Hypertrophied Spleen; Local Anæsthesia. 473. Application for the Removal of Scars; Eczema of the Head in Children; Chronic Chills; Nausea and Vomiting in Uterine Affections, 474.

EDITORIALS AND MISCELLANEOUS.

Notice; Editorial Notices; Prof. Powell's Lecture, 475. True Progress, Moral Training in Medical Schools, 476. $100,000 Premium; The Negro with a Tail; To the Reader, 478. Reviews; Pamphlets & Books Received, 479. Receipted; Special Notices, 480.

